# Burnout among kindergarten teachers and associated factors

**DOI:** 10.1097/MD.0000000000030786

**Published:** 2022-09-23

**Authors:** Syunsaku Ishibashi, Akiko Tokunaga, Susumu Shirabe, Yuri Yoshida, Akira Imamura, Kousuke Takahashi, Kojiro Kawano, Ryoichiro Iwanaga, Goro Tanaka

**Affiliations:** a Department of Occupational Therapy Sciences, Nagasaki University Graduate School of Biomedical Sciences, Sakamoto, Nagasaki, Japan; b Saikai Hospital, Gonjojimachi, Sasebo, Japan; c Department of Health Sciences, Nagasaki University Graduate School of Biomedical Sciences, Sakamoto, Nagasaki, Japan; d Center for Child Mental Health Care and Education, Nagasaki University, Sakamoto, Nagasaki, Japan; e National Research Center for the Control and Prevention of Infectious Diseases, Nagasaki University, Sakamoto, Nagasaki, Japan; f Faculty of Education, Nagasaki University, Bunkyo, Nagasaki, Japan; g Tikumaso Mental Hospital, Chuouhigashi, Ueda, Nagano.

**Keywords:** burnout, coping behavior, kindergarten teacher, social support, stress

## Abstract

Burnout among kindergarten teachers is a subject of great concern. Although burnout is reported to be significantly associated with turnover intention and work engagement, few studies have examined factors associated with burnout among these teachers. Therefore, in the present study, we performed a cross-sectional survey of burnout and associated factors among kindergarten teachers. We distributed 3363 questionnaires to all 205 authorized kindergartens and childcare institutions in Nagasaki Prefecture and received 1086 responses (response rate: 32.3%). The participants were limited to full-time female class teachers. After excluding survey forms with incomplete content, we ultimately examined valid responses from 442 participants. The survey examined burnout (Maslach Burnout Inventory, MBI), teacher stress (Nursery Teacher’s Stress Scale, NTSS), coping behaviors (Brief Scales for Coping Profile, BSCP), and social support (Social Support Scale, SSS). A multiple regression analysis revealed that all 3 MBI subscales were positively associated with “understanding of how to handle children” on the NTSS and negatively associated with “superiors” on the SSS. “Emotional exhaustion” was significantly associated with “interpersonal relations at work” and “lack of time” on the NTSS, and “avoidance and suppression” on the BSCP. “Depersonalization” was significantly associated with age, “disconnect in working conditions” on the NTSS, and “proactive problem solving” and “venting emotions to others” on the BSCP. “Diminished professional accomplishment” was significantly associated with age, “lack of time” on the NTSS, and “proactive problem solving” and “change in perspective” on the BSCP. These results suggest that support from superiors that enables teachers to better understand children and handle them appropriately is necessary to prevent burnout.

## 1. Introduction

Kindergarten teaching, a profession that involves the care and education of children up to the age of 6 years (before the start of elementary school), plays a crucial role in social development and health promotion.^[1]^ However, this role requires physical and mental effort.^[2]^ The responsibility and frustration entailed by daily duties render kindergarten teaching one of the most stressful professions^[1]^ and frequently lead to physical and mental exhaustion.^[3]^ In light of this situation, the Japanese Ministry of Education, Culture, Sports, Science and Technology (2020) has emphasized the recruitment and training of kindergarten teachers as urgent priorities.^[4]^

The term “burnout” is commonly used to express a state of mental exhaustion.^[5]^ However, burnout gradually develops over a long period of time without the person being aware of it,^[6]^ and is a risk factor for depression.^[7]^ Additionally, the World Health Organization defines burnout as a “syndrome conceptualized as resulting from chronic workplace stress that has not been successfully managed.”^[8]^ It is characterized by feelings of energy depletion or exhaustion, increased mental distance from one’s job, or feelings of negativism or cynicism related to one’s job, and reduced professional efficacy.^[8]^

Several previous studies have examined burnout and/or mental health among kindergarten teachers. For example, studies conducted in Croatia and China have reported that roughly half of kindergarten teachers experience burnout.^[1,9]^ However, in another study, the overall burnout level of preschool teachers was relatively low.^[2]^ Factors associated with burnout include teachers’ happiness at school,^[10]^ self-esteem,^[10]^ job satisfaction,^[10]^ kindergarten type,^[11]^ working hours per day,^[11]^ educational background,^[11]^ organizational climate,^[12]^ and support from fellow teachers.^[13]^ While Zhai et al^[14]^ reported that kindergarten teachers may feel stress due to a combination of factors such as handling children’s problem behaviors, low pay, long working hours, and dearth of support, their intervention study with 90 kindergarten teachers succeeded in reducing teachers’ stress by equipping them with suitable skills for handling children’s problem behaviors.

In a study with Japanese kindergarten teachers,^[15]^ more than 60% of participants exceeded the cutoff score of 5 on the Kessler Screening Scale for Psychological Distress; this percentage was similar to that reported in studies conducted with Japanese nurses.^[16,17]^ Therefore, kindergarten teachers face high levels of stress similar to those experienced by healthcare workers, and require a support system. In their study of Japanese preschool workers’ willingness to continue working, Tayama et al^[18]^ reported that gender, age, mental health, social support, and work engagement were associated with willingness to continue working. However, the study by Tayama et al included a broad range of preschool workers from part-time workers with fixed-term contracts to administrative employees; thus, there remained the need to conduct a study limited to class teachers, who have the most central role among kindergarten teachers. Therefore, Matsuo et al^[19]^ investigated willingness to continue working among full-time Japanese kindergarten teachers not in administrative positions, and found that older age, living with a spouse, caring for younger children (up to 2 years old) at work, having good mental health, and higher work engagement were significantly positively associated with teachers’ willingness to continue working.

Burnout is also reported to be significantly associated with turnover intention and work engagement.^[20,21]^ Although the recruitment and establishment of kindergarten teachers^[4]^ also renders burnout a matter of great concern,^[22,23]^ few studies have examined factors associated with burnout among these teachers.

Therefore, in the present study, we cross-sectionally surveyed burnout and factors conceivably associated with it (stress, coping behaviors, and social support) among kindergarten teachers.

## 2. Subjects and Methods

### 2.1. Participants

**Figure 1.**
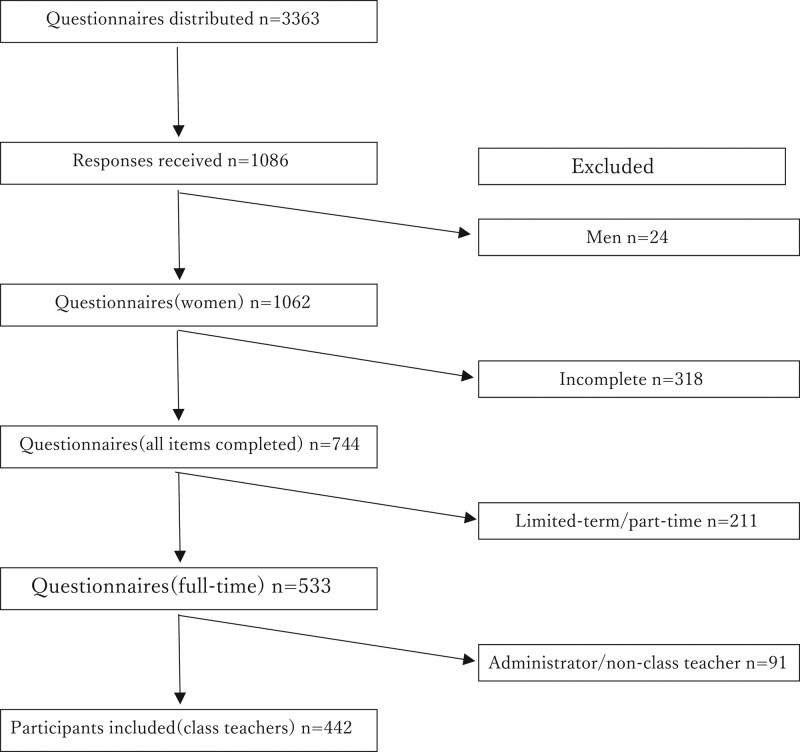
Flowchart of the participants included in the study.

The participants were recruited from all kindergartens and authorized childcare institutions in Nagasaki, Japan. A total of 3363 survey forms were distributed to all 205 kindergartens and authorized childcare institutions in Nagasaki Prefecture. The study was conducted from August to October 2019. All questionnaire responses were self-reported and provided anonymously; the participants returned their answered questionnaires in sealed envelopes. A total of 1086 responses (32.3%) were received. Burnout also varies by gender and employment status,^[24]^ while responsibility among childcare staff differs greatly depending on whether a teacher is a class teacher^[25]^; therefore, we limited participants to full-time female class teachers. After excluding survey forms with incomplete responses, we ultimately examined valid responses from 442 participants (13.1%) (Fig. 1). Informed consent was obtained from all participants before the study. The study was conducted at the Center for Child Mental Health Care and Education, Nagasaki University; was approved by the Ethics Committee of Graduate School of Biomedical Sciences, Department of Health, Nagasaki University (approval No.21102201); and complied with the Declaration of Helsinki.^[26]^

### 2.2. Measures

#### 2.2.1. Maslach burnout inventory (MBI)

Burnout was measured using the MBI.^[27]^ The MBI comprises 3 subscales: “emotional exhaustion,” “depersonalization,” and “professional accomplishment.” For example, “I feel burned out from my work” is a part of the emotional exhaustion subscale, “I worry that this job is hardening me emotionally” is a part of the depersonalization subscale, and “I have accomplished many worthwhile things in this job” is a part of the professional accomplishment subscale. Responses are provided on a 7-point scale ranging from “never” (0 points) to “every day” (6 points). The MBI consists of 22 items, with emotional exhaustion, depersonalization, and professional accomplishment addressed by 9, 5, and 8 items, respectively. Higher scores for emotional exhaustion and depersonalization represent more of those experiences. Professional accomplishment is reverse scored, meaning that a higher score represents lower professional accomplishment. Therefore, “professional accomplishment” is hereafter notated as “diminished professional accomplishment.” For the subjects in the present study, the Cronbach’s α coefficients for emotional exhaustion, depersonalization, and diminished professional accomplishment were 0.854, 0.719, and 0.793, respectively.

#### 2.2.2. Nursery teacher’s stress scale (NTSS)

The NTSS is a scale for assessing occupational stress among nursery school teachers and other childcare staff.^[28]^ The scale consists of 29 items across 6 factors: “understanding of how to handle children” (example: not understanding how children feel) (10 items), “interpersonal relationships at work” (example: attitude and behavior of other teachers) (6 items), “relationships with guardians” (example: teachers’ feelings not being conveyed to guardians) (5 items), “lack of time” (example: lack of personal time) (4 items), “disconnects in working conditions” (example: insufficient pay) (2 items), and “disconnects in school policies” (example: school rules that the participant cannot accept) (2 items). Items were rated on a 5-point scale ranging from “not true at all” (1 point) to “very true” (5 points), with higher scores representing more experiences of that stress factor. For the subjects in the present study, the Cronbach’s α coefficients for understanding of how to handle children, interpersonal relationships at work, relationships with guardians, lack of time, disconnects in working conditions, and disconnects in childcare policies were 0.888, 0.886, 0.860, 0.754, 0.769, and 0.797, respectively.

#### 2.2.3. Brief scales for coping profile (BSCP)

The BSCP is a scale that measures behaviors for coping with stressful conditions.^[29]^ The scale consists of 18 items across 6 subscales: “proactive problem solving” (example: attempting to solve a problem by examining its cause) (3 items), “consulting about solutions” (example: consulting with someone you can trust about solutions to problems) (3 items), “recreation” (example: distracting yourself with hobbies and entertainment) (3 items), “venting emotions to others” (example: having someone listen to your complaints even if you know it cannot solve your problem) (3 items), “avoidance and suppression” (example: running away from or putting off problems) (3 items), and “change in perspective” (example: considering only the positive side of an incident) (3 items). Items were rated on a 4-point scale ranging from “almost never” (1 point) to “often” (4 points), with higher scores representing more frequent engagement in that coping behavior. For the subjects in the present study, Cronbach’s α coefficients for proactive problem solving, consulting about solutions, recreation, venting emotions to others, avoidance and suppression, and change in perspective were 0.809, 0.783, 0.861, 0.803, 0.723, and 0.698, respectively.

#### 2.2.4. Social support scale (SSS)

In the present study, we used the social support subscale of the Brief Scales for Job Stress.^[30]^ The SSS, which provides a picture of how easy it is to obtain social support, consists of 3 items related to superiors, coworkers, and family/friends: “ability to speak casually,” “reliable in times of trouble,” and “listens when I consult with them about personal problems.” Items were rated on a 4-point scale ranging from “not at all” (1 point) to “very much so” (4 points), with higher scores representing greater established support. The original version of the SSS^[28]^ calculated scores for 3 categories of people: superiors, coworkers, and family/friends. The present study, however, divided family and friends into 2 separate categories, defined “family” as “spouse/family,” and calculated scores for 4 groups of people (superiors, coworkers, spouse/family, and friends). For the subjects in the present study, Cronbach’s α coefficients for superiors, coworkers, spouse/family, and friends were 0.817, 0.818, 0.836, and 0.858, respectively.

#### 2.2.5. Sociodemographic characteristics

We collected data concerning age, duration of employment, and educational level.

### 2.3. Statistical analysis

First, we used Spearman’s rank correlation coefficient to examine the correlations of basic characteristics (age group) and the scales used in the present study with the MBI subscales. Next, to examine factors associated with burnout, we used multiple regression analysis (stepwise regression) with the 3 MBI subscales as dependent variables and other survey items as independent variables. We used SPSS (version 22.0, IBM) statistical software and a statistical significance level of 5%.

## 3. Results

Table 1 shows the results for basic characteristics and assessment scales. Roughly half of the participants were in their twenties, while roughly 40% of the participants had ≥ 10 years of experience. As for educational background, roughly 70% of the participants had graduated from a vocational school, technical college, or junior college.

**Table 1:** Subjects’ characteristics (n = 442).

		n	%
Age			
	20-29	235	53.2
	30-39	111	25.1
	40-49	64	14.5
	50-59	32	7.2
Yr of work experience			
	<3	86	19.5
	3-<6	90	20.4
	6-<10	84	19.0
	≧10	182	41.2
Education			
	junior college	289	65.4
	university	150	33.9
	graduate school	3	0.7
		Mean	SD
Maslach burnout inventory			
Emotional exhaustion		26.3	10.5
Depersonalization		3.8	4.7
Professional accomplishment		19.9	8.8
Nursery teacher’s stress scale			
Understanding of how to handle children		28.8	7.9
Interpersonal relationships at work		18.1	5.8
Relationships with guardians		15.1	4.4
Lack of time		13.9	3.9
Disconnects in working conditions		6.5	2.3
Disconnects in school policies		5.2	2.3
Brief scales for coping profile			
Proactive problem solving		9.5	2.0
Consulting about solutions		9.3	2.2
Recreation		8.7	2.7
Venting emotions to others		4.1	1.7
Avoidance and suppression		5.9	2.2
Change in perspective		7.6	2.2
Social support scale			
Superiors		8.1	2.2
Coworkers		9.0	2.2
Spouse/family		10.2	2.1
Friends		9.9	2.1

Table 2 shows the correlations of the MBI with age group and scales. A correlation analysis revealed that all 3 MBI subscales were significantly correlated with age, “understanding of how to handle children” on the NTSS, 2 BSCP subscales (“consulting about solutions” and “venting emotions to others”), and “superiors” on the SSS.

**Table 2:** Correlations of the MBI with characteristics and scales.

	Emotional exhaustion	Depersonalization	Personal accomplishment
Age	-0.099*	-0.184**	-0.158**
Yr of work experience	-0.052	-0.085	-0.120*
Education	0.129**	0.083	-0.052
Nursery teacher’s stress scale			
Understanding of how to handle children	0.426**	0.469**	0.268**
Interpersonal relationships at work	0.451**	0.390**	0.088
Relationships with guardians	0.310**	0.293**	0.055
Lack of time	0.533**	0.303**	-0.010
Disconnects in working conditions	0.386**	0.327**	-0.010
Disconnects in school policies	0.393**	0.311**	0.050
Brief scales for coping profile			
Proactive problem solving	0.013	-0.207**	-0.347**
Consulting about solutions	-0.135**	-0.187**	-0.194**
Recreation	-0.067	-0.008	-0.019
Venting emotions to others	0.190**	0.287**	0.130**
Avoidance and suppression	0.213**	0.235**	0.085
Change in perspective	-0.009	-0.032	-0.289**
Social support scale			
Superiors	-0.314**	-0.333**	-0.202**
Coworkers	-0.245**	-0.261**	-0.109*
Spouse/family	-0.090	-0.161**	-0.134**
Friends	-0.062	-0.146**	-0.072

Spearman, MBI = Maslach Burnout Inventory.

* *P* < .05,

** *P* < .01.

Table 3 shows the multiple regression analysis results. All 3 MBI subscales were positively associated with “understanding of how to handle children” on the NTSS and negatively associated with “superiors” on the SSS.

**Table 3:** Results of regression analysis for MBI.

	Emotional exhaustion		Depersonalization		Personal accomplishment	
	β	*P*	β	*P*	β	*P*
Age			-0.117	.004*	-0.085	.046*
Nursery teacher’s stress scale						
Understanding of how to handle children	0.182	<.001**	0.276	<.001**	0.221	<.001**
Interpersonal relationships at work	0.175	<.001**				
Lack of time	0.331	<.001**			-0.149	.002**
Disconnects in working conditions			0.181	<.001**		
Brief scales for coping profile						
Proactive problem solving			-0.094	.022*	-0.244	<.001**
Venting emotions to others			0.152	<.001**		
Avoidance and suppression	0.091	.019*				
Change in perspective					-0.200	<.001**
Social support scale						
Superiors	-0.091	.031*	-0.171	<.001**	-0.160	<.001**
*R* ^2^	0.396		0.317		0.240	

Stepwise multiple regression analysis using subscales of MBI as dependent variables.

MBI = Maslach burnout inventory.

* *P* < .05,

** *P* < .01.

“Emotional exhaustion” was significantly associated with “interpersonal relations at work” (β = 0.182) and “lack of time” (β = 0.331) on the NTSS, and “avoidance and suppression” (β = 0.091) on the BSCP. “Depersonalization” was significantly associated with age (β = −0.117), “disconnect in working conditions” on the NTSS (β = 0.181), and “proactive problem solving” (β = −0.094) and “venting emotions to others” (β = −0.152) on the BSCP. “Diminished professional accomplishment” was significantly associated with age (β = −0.085), “lack of time” on the NTSS (β = −0.149), and “proactive problem solving” (β = −0.244) and “change in perspective” (β = −0.200) on the BSCP.

## 4. Discussion

The present study examined burnout and associated factors in full-time female kindergarten class teachers in Nagasaki Prefecture.

First, a correlation analysis revealed that all 3 MBI subscales were positively correlated with age, “understanding of how to handle children” on the NTSS, 2 BSCP subscales (“consulting about solutions” and “venting emotions to others”), and “superiors” on the SSS. Thus, factors such as young age, difficulty in understanding how to handle children, venting emotions to others without consulting about solutions, and weak support from superiors were significantly correlated with high burnout.

Next, in the multiple regression analysis, all 3 MBI subscales were positively associated with “understanding of how to handle children” on the NTSS and negatively associated with “superiors” on the SSS. “Understanding of how to handle children” on the NTSS is composed of 10 items such as, “I’m not good at handling children who concern me” and “I don’t know what kind of support children need.” Difficulty in handling children or finding it difficult to understand how to handle them are believed to increase stress^[26]^; our results in this regard are consistent with those of a previous study.^[31]^ In addition, previous studies conducted with healthcare personnel^[32–34]^ have reported that support and respect from superiors prevents burnout among organizational members. These findings suggest that support from superiors that deepens understanding of children and enables suitable care of children is necessary for preventing burnout among kindergarten teachers.

In a previous study that examined burnout among 688 Chinese kindergarten teachers using the MBI, Li Y et al^[11]^ performed between-groups comparisons, which revealed significant differences in kindergarten type (public vs private), daily working hours (<10 vs ≥10), and educational background (technical secondary school, associate degree, bachelor’s degree, or master’s degree). In a study of 436 Chinese kindergarten teachers conducted by Ji et al,^[12]^ correlation and mediating effect analysis showed that kindergarten organizational climate was negatively correlated with burnout. In a study of 1795 Chinese preschool teachers, Li S et al^[9]^ conducted a multivariate analysis, which showed that burnout was significantly associated with body mass index, type of school, income satisfaction, depression, and perceived stress levels.

Next, let us look at previous studies that used burnout scales other than the MBI. In a study of 48 Chinese kindergarten teachers in which Cheuk et al^[13]^ used an original depersonalization scale, a hierarchical regression analysis revealed a positive association between being spurned (being rejected by fellow teachers) and depersonalization. In their study of 81 kindergarten teachers and 113 elementary school teachers in Italy using the Copenhagen Burnout Inventory,^[35]^ De Stasio et al^[10]^ reported that a multiple regression analysis showed the following results: personal burnout was associated with well-being at school and self-esteem; work-related burnout was associated with happiness at school, job satisfaction, and self-esteem; and student-related burnout was associated with job satisfaction, happiness at school, self-esteem, and self-efficacy. In their study of 297 Turkish preschool teachers that used a burnout scale developed by Pines et al,^[36]^ Tornuk et al^[2]^ reported that a multivariate analysis showed a significant correlation between burnout and social support; in addition, the professional social support perceptions of preschool teachers were a significant predictor of burnout.

As described above, although previous studies conducted with participants from different cultures using different burnout scales have yielded a variety of results, some of these results support those of the present study.

We proceed to discuss specific ways of applying the results of the present study to recruit and establish kindergarten teachers.^[4]^ We determined in a previous study^[37]^ that in addition to worrying in terms of behavior, interpersonal relationships, and emotion about how to handle children who pose problems, many kindergarten teachers also worry about their relationships with superiors, coworkers, and guardians. In addition to this handling of children, the burden imposed by interpersonal relationships in the workplace cannot be ignored. Furthermore, as the free responses in the present study indicate,^[38]^ roughly 50% of the participants desired simplification of records and documents to reduce overtime. What is needed to combat burnout among kindergarten teachers is to foster a culture of dialogue in which teachers can casually discuss their concerns and desires with superiors and coworkers and share things with each other in the workplace, and to construct an education/support system for new teachers to impart the technical skills necessary for teaching kindergarten children and provide them prospects for furthering their careers.

There were several limitations to the present study. As the survey was cross-sectional, a causal relationship between burnout, stress, coping, and social support could not be established. Future empirical longitudinal studies are required to establish causality. Further, all data were collected by self-report questionnaires; thus, the possibility of self-report bias cannot be excluded. In addition, all participants in our study were female. As society develops, the number of male kindergarten teachers is growing; therefore, future studies should incorporate data from male kindergarten teachers.

## 5. Conclusions and Implications

Burnout among full-time Japanese female kindergarten class teachers was associated with stress in handing children and with support from superiors. Our results suggest that support from superiors that enables teachers to better understand children and deal with them appropriately is necessary to prevent burnout.

## Acknowledgments

The authors would like to express their appreciation to the participants of this study. This study was supported by the Center for Child Mnetal Health Care and Education, and Nagasaki University.

## Author contributions

**Conceptualization:** Syunsaku Ishibashi, Akiko Tokunaga, Susumu Shirabe, Yuri Yoshida, Akira Imamura, Kousuke Takahashi, Kojiro Kawano, Ryoichiro Iwanaga, Goro Tanaka.

**Data curation:** Syunsaku Ishibashi, Akiko Tokunaga.

**Formal analysis:** Syunsaku Ishibashi.

**Investigation:** Syunsaku Ishibashi, Akiko Tokunaga, Goro Tanaka.

**Methodology:** Syunsaku Ishibashi, Akiko Tokunaga.

**Resources:** Syunsaku Ishibashi, Akiko Tokunaga.

**Supervision:** Susumu Shirabe, Yuri Yoshida, Akira Imamura, Kousuke Takahashi, Kojiro Kawano, Ryoichiro Iwanaga, Goro Tanaka.

**Writing – original draft:** Syunsaku Ishibashi.

## References

[1] Hozo E Sucic G Zaja I. Burnout syndrome among educators in pre-school institutions. Material Socio-Medica. 2015;27:399–403. 10.5455/msm.2015.27.399-403 PMC4733556 26889099

[2] Tornuk N Gunes DZ. Perception of professional social support as a predictor of burnout level of pre-school teachers. Int J Curriculum Instruction. 2020;12:105–14.

[3] McMullen MB Krantz M. Burnout in day care workers: the effects of learned helplessness and self-esteem. Child Youth Care Quarterly. 1988;17:275–80.

[4] Ministry of Education, Culture, Sports, Science and Technology. Guidebook for the recruitment and flourishing of kindergarten teachers. Ministry of Education, Culture, Sports, Science and Technology. 2020. Available at: https://www.mext.go.jp/content/20200515-mxt_youji-100003107_01.pdf. [Access date November 3, 2021].

[5] Schaufeli WB Bakker AB. Job demands, job resources and their relationship with burnout and engagement: a multi-sample study. J Org Behav. 2004;25:293–315.

[6] Rothman S. Burnout, psychological strengths and coping strategies of senior managers in a manufacturing organization. Manage Dynamics. 2004;13:26–37.

[7] Puranitee P Saetang S Sumrithe S. Exploring burnout and depression of Thai medical students: the psychometric properties of the Maslach burnout inventory. Int J Med Educ. 2019;10:223–9. 31786565 10.5116/ijme.5dc6.8228 PMC7252444

[8] World Health Organization. Burn-out an “occupational phenomenon”: international classification of diseases. 2019. Available at: https://www.who.int/news/item/28-05-2019-burn-out-an-occupational-phenomenon-international-classification-of-diseases. [Access date September 1, 2021].

[9] Li S Li Y Lv H. The prevalence and correlates of burnout among Chinese preschool teachers. BMC Public Health. 2020;20:160. 32013939 10.1186/s12889-020-8287-7 PMC6998270

[10] De Stasio S Fiorilli C Benevenelumsa P. Burnout in special needs teachers at kindergarten and primaryschool: investigating the role of personal resources and work wellbeing. Psychol Schools. 2017;54:472–86.

[11] Li Y. Investigation on the job burnout of kindergarten teachers. Int J Front Soc. 2020;2:89–97.

[12] Ji D Yue Y. Relationship between kindergarten organizational climate and teacher burnout: work-family conflict as a mediator. Front Psychiatry. 2020;11:408. 10.3389/fpsyt.2020.00408 PMC7242754 32499727

[13] Cheuk WH Wong KS. Depersonalization in kindergarten teachers in relation to rejection of help by fellow teachers and emotional support from family. Psychol Rep. 1998;83:939–42. 9923171 10.2466/pr0.1998.83.3.939

[14] Zhai F Raver CC Li-Grining C. Classroom-based interventions and teachers’ perceived job stressors and confidence: evidence from a randomized trial in head start settings. Early Child Res Q. 2011;26:442–52. 21927538 10.1016/j.ecresq.2011.03.003 PMC3172132

[15] Yaginuma-Sakurai K Tsuno K Yoshimasu K. Psychological distress and associated factors among Japanese nursery school and kindergarten teachers: a cross-sectional study. Industrial Health. 2020;58:530–8. 32713895 10.2486/indhealth.2020-0052 PMC7708741

[16] Kunie K Kawakami N Shimazu A. The relationship between work engagement and psychological distress of hospital nurses and the perceived communication behaviors of their nurse managers: a cross-sectional survey. Int J Nurs Stud. 2017;71:115–24. 28391107 10.1016/j.ijnurstu.2017.03.011

[17] Kikuchi Y Nakaya M Ikeda M. Relationship between depressive state, job stress and sense of coherence among female nurses. Indian J Occup Environ Med. 2014;18:32–5. 25006315 10.4103/0019-5278.134959 PMC4083521

[18] Tayama J Yoshida Y Iwanaga R. Factors associated with preschool workers’ willingness to continue working. Medicine. 2018;97:e13530. 30544456 10.1097/MD.0000000000013530 PMC6310558

[19] Matsuo M Tanaka G Tokunaga A. Factors associated with kindergarten teachers’ willingness to continue working. Medicine. 2021;100:e27102. 34477148 10.1097/MD.0000000000027102 PMC8415944

[20] Gabel SR Dolan SL Suarez CA. Burnout and engagement as mediators in the relationship between work characteristics and turnover intentions across two Ibero-American nations. Stress Health. 2016;32:597–606. 26680339 10.1002/smi.2667

[21] Fragoso ZL Holcombe KJ McCluney CL. Burnout and engagement: relative importance of predictors and outcomes in two health care worker samples. Workplace Health Saf. 2016;64:479–87. 27282979 10.1177/2165079916653414

[22] Piperac P Todorovic J Terzic-Supic Z. The validity and reliability of the copenhagen burnout inventory for ezamination of burnout among preschool teachers in Serbia. Int J Environ Res Public Health. 2021;18:6805. 34202911 10.3390/ijerph18136805 PMC8297089

[23] Ma Y Wang F Cheng X. Kindergarten teachers’ mindfulness in teaching and burnout: the mediating role of emotional labor. Mindfulness. 2021;12:722–9.

[24] Pavlakis A Raftopoulos V Theodorou M. Burnout syndrome in Cypriot physiotherapists: a national survey. BMC Health Service Res. 2010;10:63. 10.1186/1472-6963-10-63 PMC2842269 20222948

[25] Zhang X Sun J. The reciprocal relations between teachers’ perceptions of children’s behavior problems and teacher–child relationships in the first preschool year. J Genet Psychol. 2011;172:176–98. 21675546 10.1080/00221325.2010.528077

[26] World Medical Association. World medical association declaration of Helsinki: ethical principles for medical research involving human subjects. JAMA. 2013;310:2191–4. 24141714 10.1001/jama.2013.281053

[27] Maslach C Jackson SE Leiter MP. Maslach Burnout Inventory Manual. 3rd ed. Palo Alto, CA: Consulting Psychologists Press. 1996.

[28] Akada T. Nursery teacher’s steress scale (NTSS): reliability and validity. Jpn J Psychol. 2010;81:158–66. 10.4992/jjpsy.81.158 20597360

[29] Kageyama T Kobayashi T Kawashima M. Development of the brief scales for coping profle (BSCP) for workers: basic information about its reliability and validity. Sangyo Eiseigaku Zasshi. 2004;46:103–14. 15382710 10.1539/sangyoeisei.46.103

[30] Okuno Y Banaba I Aono A. Sense of self-growth and burnout in hospital nurses. Jpn J Health Psychol. 2013;26:95–107.

[31] Friedman-Krauss AH Raver CC Neuspiel JM. Chile behavior problems, teacher executive functions, and teacher stress in head start classrooms. Early Educ Dev. 2014;25:681–702. 28596698 10.1080/10409289.2013.825190 PMC5460986

[32] Kanste O Kynga H Nikkila J. The relationship between multidimensional leadership and burnout among nursing staff. J Nurs Manage. 2007;15:731–9. 10.1111/j.1365-2934.2006.00741.x 17897150

[33] Lee E Esaki N Kim J. Organizational climate and burnout among home visitors: testing mediating effects of empowerment. Chilren Youth Serv Rev. 2013;35:594–602.

[34] Boamah SA Read EA Spence Laschinger HK. Factors influencing new graduate nurse burnout development, job satisfaction, and patient care quality: a time-lagged study. J Adv Nurs. 2016;73:1182–95. 27878844 10.1111/jan.13215

[35] Kristensen TS Borritz M Villadsen E. The copenhagen burnout inventory: a new tool for the assessment of burnout. Work Stress. 2005;19:192–207.

[36] Pines AM Aronson E. Career Burnout: Causes and Cures. New York: Free Press. 1988.

[37] Center for Child Mental Health Care and Education, Nagasaki University. 2018-19 report on the kindergarten teacher staff recruitment and support project. 2019. http://www.cme.nagasaki-u.ac.jp/pdf/2018jigyouhoukoku.pdf. [Access date November 3, 2021].

[38] Center for Child Mental Health Care and Education, Nagasaki University. 2019-20 report on the kindergarten teacher staff recruitment and support project. 2020. http://www.cme.nagasaki-u.ac.jp/pdf/2019jigyouhoukoku.pdf. [Access date November 3, 2021].

